# Experimental and Computational Synthesis of TiO_2_ Sol–Gel Coatings

**DOI:** 10.1021/acs.langmuir.4c03959

**Published:** 2025-01-02

**Authors:** Emőke Albert, Péter Basa, Bálint Fodor, Zsófia Keresztes, János Madarász, Péter Márton, Dániel Olasz, Adél Sarolta Rácz, György Sáfrán, Tamás Szabó, Borbála Tegze, Tibor Höltzl, Zoltán Hórvölgyi

**Affiliations:** † Department of Physical Chemistry and Materials Science, Budapest University of Technology and Economics, Műegyetem rkp. 3, 1111 Budapest, Hungary; ‡ Semilab Semiconductor Physics Laboratory Co. Ltd., Prielle Kornélia utca 2, 1117 Budapest, Hungary; § HUN-REN Research Centre for Natural Sciences, Institute of Materials and Environmental Chemistry, Magyar Tudósok Körútja 2, 1117 Budapest, Hungary; ∥ Department of Inorganic and Analytical Chemistry, Budapest University of Technology and Economics, Műegyetem rkp. 3, 1111 Budapest, Hungary; ⊥ HUN-REN Centre for Energy Research, Institute of Technical Physics and Materials Science, Konkoly-Thege Miklós út 29-33, 1121 Budapest, Hungary; # HUN-REN Computation Driven Chemistry Research Group, Department of Inorganic and Analytical Chemistry, Budapest University of Technology and Economics, Műegyetem rkp. 3, 1111 Budapest, Hungary; ∇ Nanomaterials Science Group, Furukawa Electric Institute of Technology, Késmárk utca 28/A, 1158 Budapest, Hungary

## Abstract

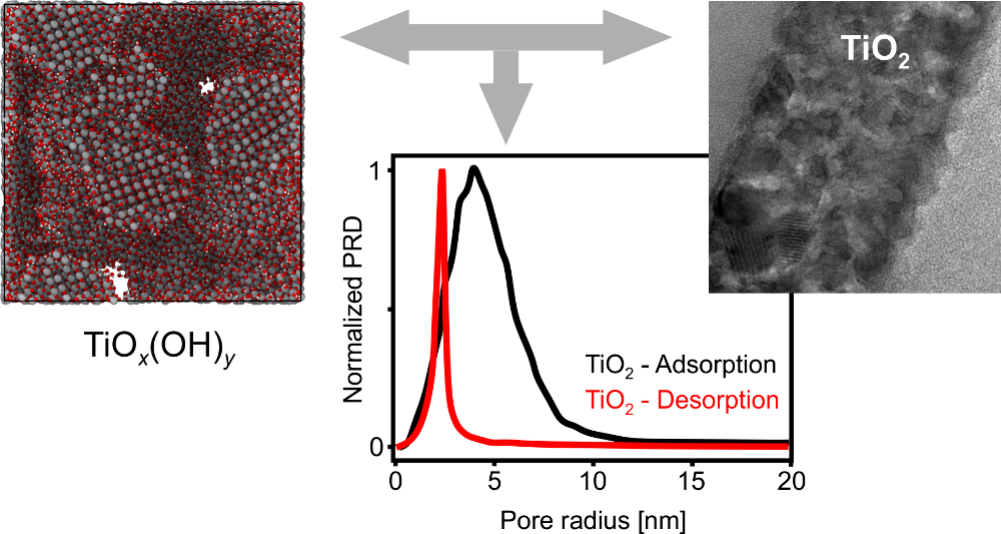

During the experimental formation of sol–gel coatings, the colloid dispersions go through a drying process, and the structure of the coatings is formed as a result of complex chemical, colloidal, and capillary interactions. While computer simulations provide guidelines to tune and even design the nanomaterials synthesis, simulations of coating structure formation are hitherto unknown in the literature. Based on real experiments, we establish here a ReaxFF reactive force field-based molecular dynamics simulation protocol in order to investigate and determine the role of the experimental conditions on the pore structure formation in the coatings. Anatase TiO_2_ sol–gel coatings with a thickness of 50 nm, 7% open porosity, and a 2.4 nm pore radius were prepared on solid substrates using the dip-coating method. In the computational synthesis of porous TiO_2_ layers, the attractive capillary forces present during the drying step were accounted for by applying an external pressure, and their effect on the coatings’ pore structure was investigated. It was found that the TiO_2_ layer structure corresponding to an external pressure of 10,000 atm in the simulations exhibited a porosity comparable to that determined by experimental methods. This demonstrates the impact of immersion capillary forces on sol–gel layer formation. The created computer model accurately describes the layer structure using real parameters, making it suitable for designing the coating structure through computer simulation.

## Introduction

Nowadays, nanomaterials with precisely defined composition and structure play a ubiquitous role in the development of novel catalysts, energy storage, or sensing. Often, the exceptional activity of these materials is achieved through controlled synthesis techniques. Nevertheless, in order to enable a rational synthesis approach and, more specifically, to propose appropriate synthesis conditions, it would be highly desirable to have an atomic-level understanding of the processes and even the ability to predict them.

Currently, computer simulations specifically designed for nanoscale reactivity are readily accessible.^1,2^ These methods are extensively applied to predict the formation and structure of carbonaceous materials, such as graphene,^3^ carbon nanotubes,^4^ functionalized amorphous carbon materials,^5^ and even the complex structure of metal nanoparticles during chemical vapor deposition on these materials.^6^ Thus, for carbonaceous materials, both the formation and the final, nanoscale structure of the materials can be simulated and predicted.

Similarly, these methods are widely employed to investigate various oxide materials, including the structure and solvation of SiO_2_,^7^ ZnO,^8^ TiO_2_^9^ or even complex oxides, such as lithium-ion conducting oxides.^10^ The atomic scale processes during the formation of these oxides at the atomic scale can also be accurately modeled,^11^ including processes like atomic layer deposition of Al_2_O_3_,^12^ and the nucleation process involved in sol–gel silica formation.^13^ However, the nanoscale structure of these oxides holds significant importance in practical applications. Compact and porous nanocoatings on solid substrates are extensively researched for their tailored optical, electrical, and mechanical properties, as well as for their photocatalytic,^14−17^ photovoltaic,^18^ anticorrosion,^19^ water-repellent,^20^ antibacterial,^21,22^ and self-cleaning^23^ behaviors. The sol–gel process offers the advantage of allowing the structure, morphology, and physicochemical properties of the coatings to be adjusted over a wide range by varying the experimental parameters. The pore system of mesoporous thin coatings can also be employed for the delivery of various nanoparticles^22^ and molecules (e.g., drug molecules, dye molecules, corrosion inhibitor molecules).^15,24,25^

TiO_2_ has been identified as a potential hazard in various fields, including the food industry, cosmetics, and biomedical applications.^26^ However, its use in light transmission regulation and photocatalytic and photovoltaic applications does not pose direct risks to human health. Moreover, the quantities required for porous nanocoating applications are minimal, with coating thicknesses typically ranging from 100 to 200 nm.

TiO_2_ is a polymorphic material, having three common phases: anatase (tetragonal), rutile (tetragonal), and brookite (orthorhombic).^27^ The formation of the preferred TiO_2_ crystalline phase depends on the starting material (e.g., composition of the precursor sol, catalyst used during sol preparation), deposition method, and the conditions, including the annealing temperature. TiO_2_ thin films can transform from the amorphous phase into crystalline anatase and from anatase into a rutile phase by calcination. It has been revealed that when a TiO_2_ thin film was subjected to a calcination temperature of 300 °C, it exhibited an amorphous structure. However, at 400 °C, it underwent a transformation into the anatase phase, and at 1000 °C, it further transitioned into the rutile phase.^28^

The tetragonally structured anatase crystal phase is the most commonly utilized form of titanium dioxide for photocatalytic applications.^27^ Utilizing titanium dioxide in its immobilized form, such as a TiO_2_ nanocoating, as a heterogeneous catalyst offers the advantage of simple and direct separation from the medium after the photocatalytic process, in contrast to when it is used in powdered form.^21^ The efficiency of photocatalytic processes can be significantly enhanced by increasing the specific surface area of the immobilized TiO_2_ photocatalyst through the application of mesoporous coatings.^29^

The sol–gel method is a preferred process for the development of TiO_2_ coatings since it is cost-effective, requires relatively simple equipment, and utilizes mild conditions, including low temperatures. The structure of the deposited thin films is determined by all the conditions applied during the precursor sol synthesis and the formation of the coating. Controlling the structure of the species present in the precursor sol, deposition and post-treatment conditions results in precise tailoring of the coatings’ structure and properties, like the porosity, surface area, pore size, and the refractive index. In addition to the composition, the size and degree of branching of the species (microphases or polymer chains) present in the precursor sol before layer deposition, as well as the relative rates of evaporation and condensation during the deposition process, also play a crucial role in controlling the structure of the coating. The layer can be deposited using different methods, such as dip-coating, spin-coating, spray-coating, etc. During the dip-coating process, microphases or polymer chains are concentrated on the surface of the solid substrate in a very short time due to the evaporation of the dispersion phase (usually ethanol).^30^ The layer thickness is determined by the competition among surface tension (capillary pressure), gravity, and viscosity. According to the Landau-Levich equation,^31^ there is a predictable correlation between the withdrawal speed and the coating thickness, even for the dip-coated sol–gel coatings prepared from precursor sols. During the final stage of the deposition process, evaporation leads to an increase in capillary pressure.^30^ As a result, the deposited film undergoes compaction primarily due to evaporation, followed by the influence of the capillary pressure. The effect of capillary pressure is much greater in thin films than in bulk gels, since greater shrinkage precedes the critical point, where the liquid–vapor interface first recedes into the gel. This results in a decreased pore size and increased maximum capillary pressure.

During the heat treatment step at elevated temperature, residual organic compounds are burned out from the as-deposited thin coating, and the crystallization of the functional oxides takes place. This step also significantly influences the surface properties and resulting pore structure, including porosity, pore size distribution, and the shape of the pores.

The significance of introducing porosity in TiO_2_ coatings is highlighted by the fact that TiO_2_, due to its high refractive index, reflects visible light when applied to transparent substrates with refractive indices similar to those of glass. According to the principles of thin-film optics, it is necessary to reduce the refractive index of TiO_2_, a process that can be achieved by introducing porosity. This leads to a decrease in the effective refractive index. Pores that are sufficiently small do not induce light scattering, which consequently reduces reflection and enhances light transmission. This is particularly important for the development of light-transmitting, photocatalytically active (self-cleaning) coatings.^32^ Furthermore, silver nanoparticles can be incorporated into the pores, resulting in coatings with antibacterial properties even in the dark.^17,22^ Such coatings would be highly advantageous as protective layers for solar panels, providing protection against weathering.

Molecular dynamics simulation methods are promising and practically important for the investigation of processes taking place during the synthesis of various nanomaterials, self-organization of molecular nanostructures, behavior of molecules in confined spaces, etc. Molecular dynamics simulations have been carried out for different polymorphs of TiO_2_ using quantum chemical methods,^33^ force fields,^34^ including ReaxFF,^35,36^ and recently using machine learning potentials,^37,33,38^ both in vacuum and also in water. Surface growth of TiO_2_ by using the sputtering technique has been investigated by joint experimental and computational (molecular dynamic) techniques.^33^ The pressure-driven transition of bulk TiO_2_ has been simulated recently by using the deep-learning molecular dynamics method.^37^ The mechanism of oriented attachment of TiO_2_ particles has been modeled by using a molecular dynamic technique,^39^ while agglomeration, annealing, and synthering of TiO_2_ particles have also been investigated in the gas phase.^40,41^ It is worth noting that the water, confined in idealistic TiO_2_ nanopores and nanoslits, has been simulated by using molecular dynamic techniques.^38,42^

However, due to the complexity of the various processes occurring during the sol–gel thin film synthesis (detailed above), which all influence the final properties of the system, predicting the resulting parameters of nanostructured TiO_2_ coatings by simulations is a serious challenge. While challenging, the development of such simulation methods is crucial for both practical applications (by potentially decreasing the necessary experimental steps when developing new TiO_2_-based nanomaterials for various uses) and for deepening the theoretical understanding of the atomic-scale processes involved in the formation of nanoporous structures in TiO_2_ (bottom-up synthesis).

In our present work, we studied whether the structure formation of sol–gel coatings can be approximated by molecular dynamics simulations, and we created a computer model and protocol suitable for predicting the coating structure. To achieve this, we defined the key parameters that determine the structure formation, such as particle size, size distribution, shape of the particles, capillary forces, etc. These parameters, determined by real experimental investigations, were used in the computer modeling program, allowing us to observe the resulting layer structure in the simulation. By comparing the simulated and real values of parameters, such as porosity, pore size, and pore size distribution, we assessed the model’s accuracy.

We observed that the model accurately describes the layer structure using real parameters, which allowed us to explore the possibility of designing the coating structure through computer simulations.

## Experimental Section

### Materials

Materials used for precursor sol synthesis: titanium(IV) butoxide (purum, ≥ 97%, Sigma-Aldrich), ethanol (EtOH, g.r., > 99.8%, Lach-Ner), nitric acid (special grade, 65%, Lach-Ner), and ultrapure water (18.2 MΩ cm, purified with an Adrona Integrity+ filtration system).

Quartz glass slides (25 × 30 × 1.05 mm, Suprasil 1, Optilab Kft., Hungary) and silicon wafers (Siegert, (100), p-type, prime grade) were used as solid substrates of the coatings. Solid substrates were cleaned using 2-propanol (2-PrOH, for analysis, ACS reagent) and ultrapure water.

2-propanol was also used as an adsorptive material in the ellipsometric porosimetry measurements.

### Synthesis of the Precursor Sol

Titania precursor sol was prepared by the acid-catalyzed controlled hydrolysis of titanium(IV) butoxide in ethanolic media. Nitric acid was used as a catalyst in the synthesis of precursor sol. The molar ratios for titanium(IV) butoxide:EtOH:HNO_3_:H_2_O were 1:27.95:0.49:0.82. The mixture was stirred for 2 h at 60 °C at 400 rpm.^43^

### Preparation of the Samples

Quartz and silicon substrates were cleaned with 2-PrOH and distilled water before layer deposition. In order to increase the adhesion and wettability of the silicon substrates by the ethanolic precursor sol, cleaned silicon substrates were also treated with O_2_ plasma (radiofrequency-generated low-pressure plasma/SmartPlasma 10, Plasma Technology GmbH/coupled with an oxygen concentrator/DeVilbiss Healthcare 525 KS/) prior to layer deposition for 10 min (0.4 mbar, 300 W).

Sol–gel coatings on solid substrates were prepared from the 1-day aged precursor sol by the dip-coating method. Clean quartz and silicon substrates were immersed into the precursor sol and pulled out with a constant speed of 12 cm/min by a dip-coater device (Plósz Mérnökiroda Kft., Hungary). The deposited films were annealed in a drying oven (Nabertherm B170) at 450 °C for 30 min with a heating rate of 5 °C/min.

Powder samples were prepared in order to model the investigated TiO_2_ coatings: 1-day aged precursor sols were dried for 2 h until the evaporation of the ethanol, and then different heat treatments were carried out. One of the samples was dried at 80 °C for 40 min, while the other sample was heat-treated by applying the same parameters as for the TiO_2_ coatings: 450 °C for 30 min with a heating rate of 5 °C/min.

### Investigation Methods

#### Transmission Electron Microscopy (TEM)

1-day aged precursor sol and the structure of the TiO_2_ coating deposited on a silicon substrate were investigated by TEM using a Thermo Fisher (Waltham, MA, USA) Titan Themis 200 kV spherical aberration (Cs)-corrected TEM/STEM microscope having 0.08 nm high-resolution TEM and 0.16 nm scanning TEM point resolution, equipped with 4 Super-X EDS detectors. Before the TEM measurement, 1 droplet of the precursor sol diluted with ethanol was deposited onto a carbon-coated copper grid, followed by a drying step under an infrared heat lamp. TiO_2_ coating on silicon substrate was prepared in cross-section for TEM investigation by conventional mechanical and ion beam thinning techniques.

#### Atomic Force Microscopy (AFM)

AFM measurements were carried out by an AIST-NT SmartSPM-1000 atomic force microscope in noncontact and tapping modes using NanoSensors PPP-NCHR AFM probes with a tip radius of less than 7 nm. Before the measurements, the 1-day aged precursor sol was diluted with ethanol to 10 times, 100 times, and 200 times its original concentration (hereafter designated as C_0_/10, C_0_/100, and C_0_/200). A 50 μL drop of these diluted sols was spin-coated onto cleaned and plasma-treated Si substrates at 6000 rpm until complete drying.

#### Dynamic Light Scattering (DLS): Size and Zeta Potential Measurements

The Z-average mean diameter, the intensity-related size distribution, the polydispersity index, and the zeta potential of the colloidal particles in the 1-day aged precursor sol were determined, based on DLS principles, using a Zetasizer Nano ZS size analyzer (Malvern Panalytical Ltd. Malvern, United Kingdom; control and data processing by Malvern software). Size and zeta potential measurements were carried out at 25 °C using a disposable folded capillary cell (DTS1070). Measurement time, number of runs, position, and attenuation were set to automatic. The zeta potential values were obtained by using Henry’s function f(κa) reduced to the Huckel limit of 1.

#### X-ray Diffraction (XRD)

The crystallinity of the TiO_2_ coatings and powder samples was characterized by an X-ray diffractometer (Philips PANalytical X’pert Pro, with Cu–Kα radiation) and an X’celerator detector. Measurements were carried out in the 2Θ = 4°–84° range, with a scanning rate of 20 s/step, step size of 0.0167°, and applying the automatic divergence slit system with an irradiated length of 15 mm.

#### X-ray Photoelectron Spectroscopy (XPS)

The surface chemical composition of the TiO_2_ coating on the Si substrate has been measured by XPS in a ThermoFisher Scientific Escalab Xi^+^ instrument. The information depth of the technique is 5–10 nm. The X-ray source was an aluminum anode (Al Kα, photon energy 1486.6 eV), and the spot size was 900 μm. The sample was fixed to the sample holder by double-sided carbon tape. As the sample was conductive enough, a charge compensation system was not applied. The survey spectrum was measured with 100 eV pass energy in steps of 0.5 eV with 10 ms dwell time per data point. Carbon (1s), oxygen (1s), silicon (2p), and titanium (2p) high-resolution spectra were taken at 20 eV pass energies with 0.1 eV steps and 50 ms dwell time per data point. As the titanium peak is very sensitive to argon sputtering,^44^ the sample was gently cleaned by an argon gas cluster source: 4 keV energy, cluster size 1000. The evaluation of the spectra was performed using Avantage software. The chemical composition was calculated from the high-resolution spectra after the background was removed with the application of the Althermo sensitivity factor library. For revealing the bonding states, the spectra were fitted by Gaussian–Lorentzian functions with a Gaussian contribution of 30%. The slight charging was corrected by setting the C–C and C–H components to 284.8 eV.

#### UV–Visible Spectroscopy (UV–Vis)

The optical properties of the coatings on quartz substrates were measured by UV–vis spectroscopy. Transmittance spectra of the bare quartz substrates and coated samples were taken using a Hanon i9 UV–vis spectrophotometer in the wavelength range of 400–1100 nm with a 1 nm resolution and a scanning speed of 10 nm/s. The obtained transmittance curves were analyzed in terms of thin-layer optical models. Transmittance spectra of coatings were fitted with a homogeneous layer model, supposing a perpendicular angle of incidence and identical homogeneous monolayers on both sides of the transparent substrate.^45^ (For further details, please refer to Section 1.1 in the Supporting Information.) The fitting procedures provided the effective refractive index (at 632.8 nm) and layer thickness values. The fitting procedure used a Levenberg–Marquardt algorithm. The porosity of coatings, based on the obtained effective refractive index values, was estimated using the Lorentz–Lorenz formula,^46^ considering the refractive index of the bulk TiO_2_ to be 2.400.^47^ (See also Section 1.3 in the Supporting Information.)

#### Scanning Angle Reflectometry (SAR)

In order to investigate the optical properties of the samples, coatings on quartz and silicon substrates were studied by SAR using p-polarized light.^48,49^ Reflectance curves of the samples were taken using a scanning angle reflectometer (Plósz Mérnökiroda Kft., Hungary). The light source was a 10 mW polarized beam He–Ne laser (Thorlabs HNL050L, with a wavelength of 632.8 nm), and the intensity of reflected light was detected using a standard photodiode power sensor (Thorlabs S120C, 50 nW–50 mW). The angle of incidence was varied by using a unipolar stepper motor (Little Step-U). Measurements were carried out around the Brewster angle of the sample-air interfaces: in the incidence range of 60°–80° for the bare Si substrate, 45°–80° for TiO_2_ coatings on Si substrate, 50°–65° for bare quartz substrate, and 50°–80° in the case of TiO_2_ coatings on quartz substrate, with a resolution of 0.01°. Due to multiple reflections of the light in the quartz substrate, a dense interference wave pattern appeared in the reflectance curves. The wave pattern was eliminated by smoothing (by adjacent averaging). Reflectance curves were evaluated by fitting them with homogeneous layer models supposing identical homogeneous monolayers on both sides (quartz) or homogeneous monolayer on one side (silicon) of the solid substrate. (For further details, please refer to Section 1.2 in the Supporting Information.) The fitting procedure used a Levenberg–Marquardt algorithm and provided the effective refractive index and thickness of the coatings. For the determination of layer thickness, an approximate value (determined by other independent methods, e.g., cross-sectional TEM) was used as the initial value in the fitting procedure.

#### Ellipsometric Porosimetry (EP)

Ellipsometric porosimetry was used to investigate the TiO_2_ coatings on quartz and silicon substrates to determine the open porosity, pore size, and pore size distribution of the samples. The measurements were carried out by a Semilab’s Sopra EP-12 ellipsometric porosimeter. EP is a technique suitable for characterizing porous thin films for open porosity, surface area, pore size, and mechanical strength. EP is a coupled technique in which the vapor adsorption can be studied step-by-step through spectroscopic ellipsometry. During the measurement, vapor of the adsorptive material condenses in the pore system, which induces an effective refractive index shift. Measuring the adsorption and the desorption isotherms, the porosity of the sample can be determined; furthermore, the pore size distribution can be calculated based on the modified Kelvin equation in the case of mesopores. The TiO_2_ coatings deposited onto quartz and silicon substrates were characterized by this method using 2-PrOH as adsorptive material. Before the measurements, the samples were heated to 448 K for 15 min and then vacuumed. The 2-propanol adsorption isotherms were measured at 297 K. EP also provides the possibility to determine the optical properties of the samples as a spectroscopic ellipsometer at the zero relative pressure of the adsorptive material. The measurements were carried out at the angle of incidence of 60° in the wavelength range of 248.5 nm–971.5 nm. The thickness and effective refractive index (at 632.8 nm) values of the coatings were determined by applying the Tauc-Lorentz oscillator model.

### Molecular Dynamics Simulations

The molecular dynamics simulations were carried out using the LAMMPS simulation program,^50^ while the results were analyzed and visualized using Ovito.^51^ The computations were carried out using the ReaxFF method.^52^ Here, we applied the force field, developed and validated for various crystal structures of titanium dioxide and the interaction of water with TiO_2_ surfaces.^35^ The TiO_2_ nanoparticle model was constructed by carving a spherical particle from a sufficiently large supercell using Python code employing the Molmod library. Solvent water molecules were inserted by using the Packmol program.^53^

The time step of 0.25 fs was applied for all simulations. Constant temperature and pressure were set using the Nosé–Hoover thermostat and barostat (including the Martina-Tuckerman-Klein formalism) with time constants of 25 and 250 ps, respectively.

All computations were carried out on Nvidia GPUs using the KOKKOS library,^54^ integrated into the LAMMPS software. Utilization of GPUs allowed for unprecedented size (ca. 73,000 atoms) and ∼ hundred ps simulation time. All simulations employ periodic boundary conditions in 3 dimensions. The details of the model system setup are described later in the article.

The pore structure was analyzed using Poreblazer.^55^ Porosities were estimated using two methods: the alpha-shape method (denoted by ε_α_; the computations use a probe radius of 8.154 Å, a sum of the van der Waals radii of the 2-propanol,^56^ and the oxygen atom,^55^ which is assumed to be present on the surface) and the Gauss-density (denoted by ε_G_; using the radius scaling of 1.0 and contour level of 0.2), as implemented in the Ovito code.^51^ As ε_α_ is parametrized for 2-propanol, we expect to model the experiments more closely. TEM images were simulated using the Prismatic code.^57^

## Results and Discussion

### Characterization of the Precursor Sol: TEM, AFM, and DLS Investigations

**Figure 1:**
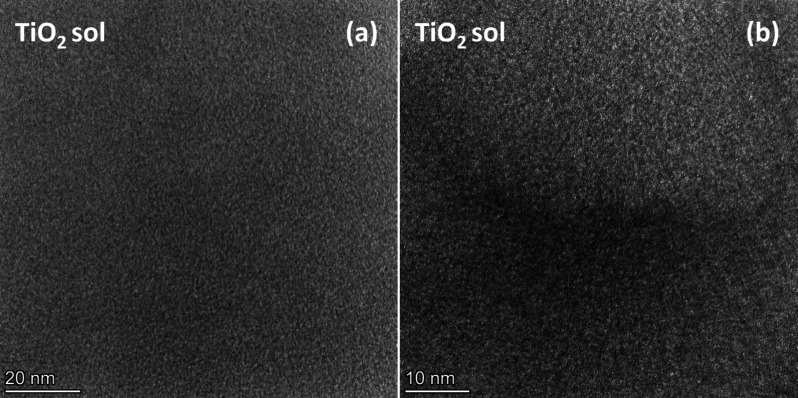
TEM images of the 1-day aged TiO_2_ precursor sol taken with lower (a) and higher (b) magnification.

The 1-day aged TiO_2_ precursor sol was investigated by TEM to obtain information about the nature and size of the species present in the sol used for layer deposition. A homogeneous layer, formed through drying from the diluted precursor sol during sample preparation for TEM measurement, can be observed; however, the presence of individual solid microphases cannot be detected in the TEM images (Figure 1a,b). The sample showed an amorphous character, with no indications of particles, either amorphous or crystalline, observed even under high resolution. Additionally, no crystalline regions were detected through electron diffraction analysis (Figure 1a,b).

Samples on Si-substrate prepared from the 1-day aged precursor sol, diluted with ethanol to C_0_/10, C_0_/100, and C_0_/200 concentrations (C_0_ is the initial concentration of the precursor sol), were also measured by AFM for the investigation of the colloidal particles present in the precursor sol.

Based on the AFM images (Figures SI1 and SI2 in the Supporting Information), it can be concluded that the dilution of the precursor sol influences the structure due to evaporation; however, individual particles are not visible. (For further details, please refer to Section 2.1 in the Supporting Information.)

The average diameter and polydispersity index value of the particles present in the ethanolic TiO_2_ precursor sol were determined by DLS. The measurement was carried out on the 1-day aged TiO_2_ sol to get information about the colloidal content of the sol used to form the coatings (as the coatings were deposited from the 1-day aged precursor sol). The representative size distribution function by the intensity of the 1-day aged TiO_2_ sol measured by DLS is shown in Figure SI3 in the Supporting Information. Z-average diameter (including the solvation shell) of approximately 2.7 nm, full width at half-maximum also of 2.7 nm, and polydispersity index of 0.098 ± 0.011 were obtained for the TiO_2_ precursor sol synthesized and stored under the same conditions for the same time as the precursor sol used for layer deposition. The DLS measurement has shown the presence of very small colloid particles with a diameter of 2.7 nm. Unfortunately, due to low-contrast features, those could not be visualized by TEM.

The identification of individual colloidal particles is further hindered by the deposition of unreacted precursor molecules and low-molecular-weight oligomers (“intermediates”) onto the TEM grid surface and onto existing larger (colloidal-sized) particles, resulting in the formation of a continuous, coherent layer. This phenomenon presents a challenge similar to that in AFM analyses. Increasing sample dilution facilitates the dissolution of increasingly larger oligomers, thereby reducing the probability of compact microphase formation. We propose that compact microphases form exclusively during the coating process as the concentration of the colloidal system increases. In DLS measurements, both larger oligomers and microphases present in varying quantities are detected collectively at a given dilution. Consequently, DLS currently serves as the sole viable method for the observation of colloidal particles (larger oligomers, macromolecules, and microphases).

The zeta potential of the 1-day-aged precursor sol was determined to be +20.30 ± 1.39 mV. In aqueous systems, the limit of colloidal stability is ±25 mV. As expected, the observed positive zeta potential can be attributed to the acidic nature of the precursor sol, coupled with the isoelectric point of anatase TiO_2_, which is reported at pH 5.8.^58^ This value suggests that the sol exhibits a moderate stability; therefore, spontaneous aggregation of colloidal particles is unfavorable, allowing the particles to remain well-dispersed within the precursor sol. This dispersion probably contributes to the formation of a homogeneous gel layer on the substrate surfaces.

### Characterization of the Powder Samples by XRD

XRD measurements were carried out on TiO_2_ coatings on silicon substrates. However, no peaks could be detected on the XRD patterns (not shown here) because of the very thin titania coatings on the substrates. In order to get better XRD results, TiO_2_ powder samples were prepared from the 1-day aged precursor sol: one of the powder samples was dried at 80 °C, and the other sample was heat-treated at 450 °C (under the same conditions as the coatings on solid substrates). XRD patterns of the powder samples prepared from the 1-day aged TiO_2_ precursor sol are presented in Figure SI4 in the Supporting Information. The sample dried at 80 °C (Figure SI4, black pattern) is an amorphous material and does not show crystallinity. In the case of the powder sample heat treated at 450 °C, all XRD peaks (Figure SI4, red pattern) are attributed to the anatase [PDF 98-000-9852] crystalline phase of TiO_2_ which was also expected based on the applied annealing temperature during sample preparation, and it is in accordance with our previous results.^22^

### Characterization of the Coatings on Solid Substrates: XPS, UV–Vis, SAR, EP, and TEM Analysis

The prepared TiO_2_ coatings exhibited homogeneity on both quartz and silicon substrates. The sample on the quartz substrate was also transparent. (Please also refer to sample images in Figure SI5 in the Supporting Information.)

According to the XPS survey spectrum (Figure SI6 in the Supporting Information) of the TiO_2_/Si sample before cluster sputtering, C, O, and Ti are present on the surface, while Si could not be detected, indicating that the coating fully covers the silicon substrate. As usual, O and Ti photoelectron peaks are also accompanied by the Auger peaks.

Table 1 presents the atomic composition results before and after cluster sputtering, demonstrating that the coating can be considered as stoichiometric TiO_2_. It should be noted that the measured carbon content mainly originates from the organic contamination, which is always present on samples exposed previously to air. Its content is strongly reduced due to cluster sputtering; however, it could not be completely removed. This might be due to the lower material removal of the applied cluster sputtering technique and that the coating might contain carbon residual due to the titanium(IV) butoxide precursor.

**Table 1:** Atomic Composition of the TiO_2_/Si Sample Surface (Before and After Cluster Sputtering) Measured by XPS

sample	O [at%]	Ti [at%]	C [at%]	O/Ti [-]
TiO_2_/Si (before cluster sputtering)	55.4	26.8	17.8	2.1
TiO_2_/Si (after cluster sputtering)	63.7	32.1	4.2	2.0

The high-resolution Ti 2p spectra before and after cluster sputtering are shown in Figure SI7 in the Supporting Information. It can be seen that the titanium splitting value (energy difference between 2p 3/2 and 1/2 peaks) which varies with chemical state, was found to be 5.7 eV, aligning well with the literature value for TiO_2_.^59^ A slight broadening of the peak on the low-energy side can also be observed after cluster sputtering. This can be attributed to reduced states of titanium,^60^ meaning that even the gentle cluster sputtering technique can cause damage in the titanium peak.

The O 1s peak could be decomposed into three components which are depicted in Figure SI8 in the Supporting Information, before (Figure SI8a) and after (Figure SI8b) sputtering. The lowest binding energy component at 530.0 eV can be ascribed to lattice oxygen (L_O_), namely, the Ti–O bonds in TiO_2_, while the components at 531.3 and 532.3 eV can be attributed to oxygen vacancy/defects (V_O_) and loosely adsorbed, dissociated oxygen or OH species (C_O_).^61^ It can be seen that compared to the sample before cluster sputtering (Figure SI8a), the rate of the C_O_ species is strongly reduced after sputtering (Figure SI8b).

**Figure 2:**
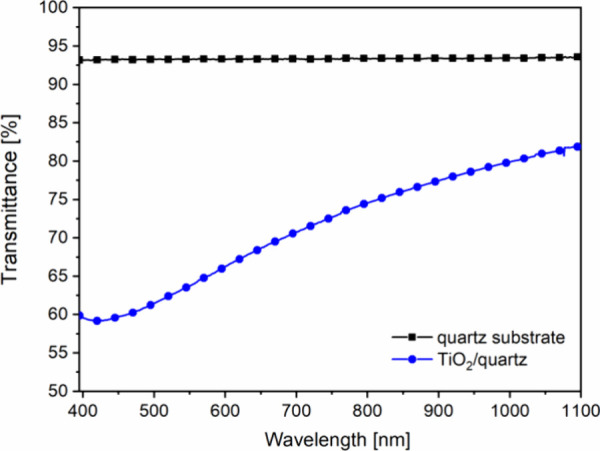
Representative transmittance spectra of the bare quartz substrate and the TiO_2_ coating on the quartz substrate.

The representative transmittance curves of the uncoated, bare quartz substrate and the TiO_2_ coating on quartz can be seen in Figure 2. The TiO_2_ coating decreases the light transmittance of the quartz substrate in the whole studied wavelength range, due to its higher effective refractive index value (see Table 2) than that of the bare quartz substrate (*n*_quartz_ = 1.4570). Thickness and effective refractive index values of the TiO_2_ sol–gel coating were determined by applying fitting procedures (Table 2). (For further details, please refer to Section 1.1 in the Supporting Information.) Porosity calculated by the Lorentz–Lorenz formula (see also Section 1.3 in the Supporting Information) in view of the effective refractive index of the coating and refractive index of the bulk TiO_2_ (*n*_bulk_ = 2.400) and air (*n*_air_ = 1) is also presented in Table 2.

**Table 2:** Effective Refractive Index, Thickness and Calculated Porosity Values of the TiO_2_ Coatings on Quartz and Silicon Substrates, Determined from the UV-Vis Transmittance Spectra (Quartz) and Reflectance Curves (Quartz and Silicon) of the Samples

coating/substrate	method	refractive index (632.8 nm) [-]	thickness [nm]	porosity (Lorentz–Lorenz) [%]
TiO_2_/quartz	UV–vis	2.0341	53	17
TiO_2_/quartz	SAR	2.0504	56	16
TiO_2_/Si	SAR	2.1698 ± 0.0051	52 ± 1	10 ± 0.25

The refractive index, thickness, and porosity values determined based on the SAR measurements are also presented in Table 2. (For further details, please refer to Section 1.2 in the Supporting Information.) The representative reflectance curves of the TiO_2_ coatings on quartz and silicon substrates and their uncoated substrates can be found in the Supporting Information (Figure SI9).

Comparing the results obtained from the transmittance curves with those derived from the reflectance curves, a good agreement in the value of the refractive index, thickness, and porosity for the TiO_2_ coating on the quartz substrate can be observed. TiO_2_ coatings prepared exactly under the same conditions on quartz and silicon substrates, however, show different refractive index and porosity values (Table 2). This can be attributed to the influence of substrate type on the structure of sol–gel coatings, a topic for which the available literature is limited, and the underlying mechanisms remain poorly understood. Only a small number of scientific studies have addressed this issue. Nikolic et al. observed that the transformation from amorphous to anatase TiO_2_ progressed more slowly on quartz compared to that on a silicon substrate. Additionally, they described the orientation of TiO_2_ coatings on silicon and explored the nature of the interactions between titania and silicon substrates.^62^

**Figure 3:**
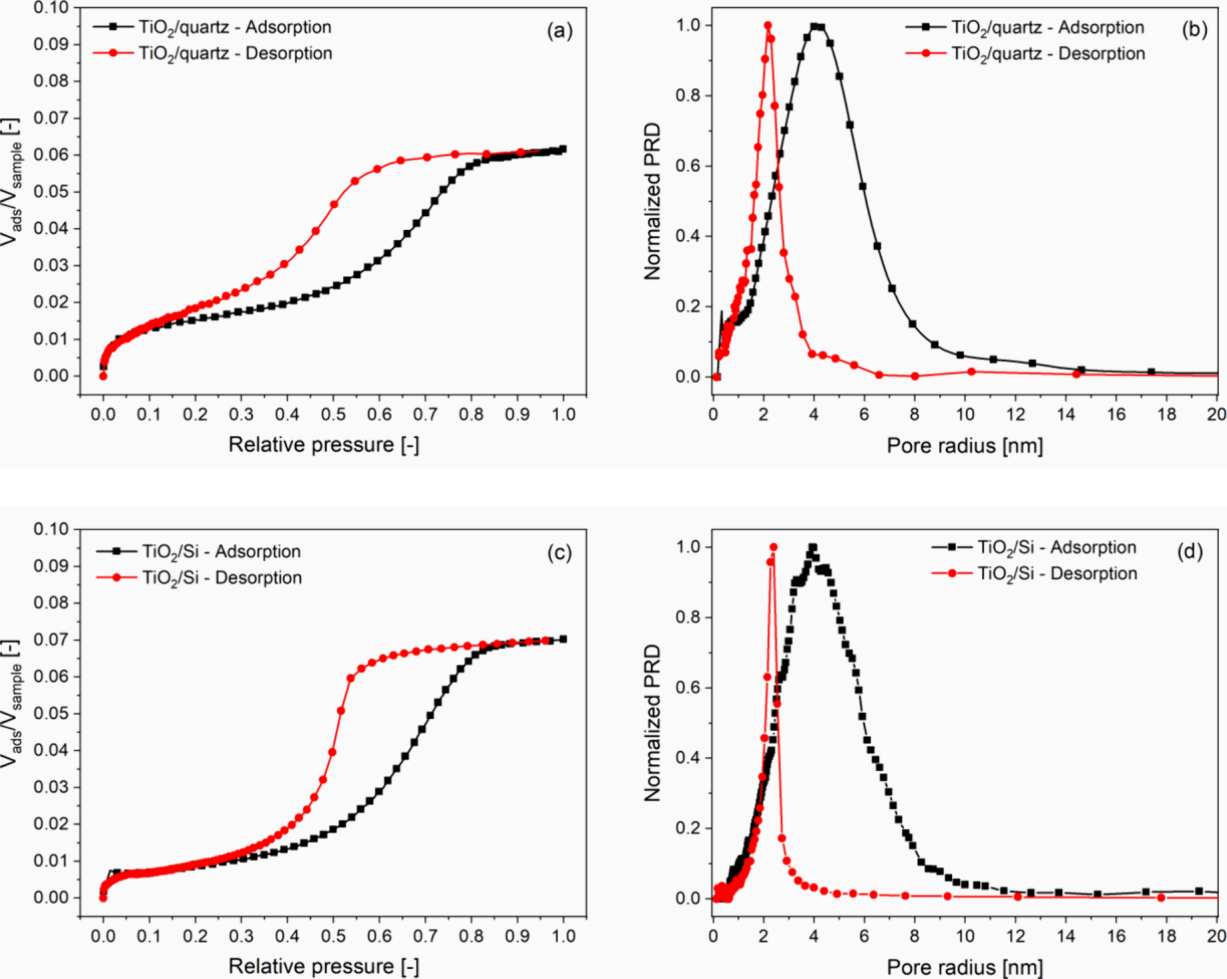
2-Propanol adsorption–desorption isotherms taken at 297 K (a,c) and normalized pore radius distribution functions (b,d) of TiO_2_ coatings on quartz (a,b) and silicon (c,d) substrates, determined by ellipsometric porosimetry.

In order to investigate the amount and character of the available open pores, the TiO_2_ coatings on quartz and silicon substrates were characterized by ellipsometric porosimetry. The adsorption–desorption isotherms shown in Figure 3a,c were taken at 297 K using 2-propanol as an adsorptive material. The normalized pore radius distributions of the pore systems presented in Figure 3b,d were determined by using the modified Kelvin equation and assuming cylindrical pores. The shape of the isotherms is of type IV, and the hysteresis loops are of type H2, according to IUPAC classification.^63^ These correspond to mesoporous materials with interconnected pore systems and nonuniform pore sizes.^64^ The effective refractive index (at 632.8 nm) and thickness values of the coatings were also determined by EP at zero relative pressure by applying the Tauc-Lorentz oscillator model (Table 3).

**Table 3:** Effective Refractive Index, Thickness, Total and Open Porosity, and Pore Radius Values of the TiO_2_ Coatings on Quartz and Silicon Substrates Determined by Ellipsometric Porosimetry

coating/substrate	refractive index (632.8 nm) [-]	thickness [nm]	total porosity (Lorentz–Lorenz) [%]	open porosity [%]	*r*_ads_ [nm]	*r*_des_ [nm]
TiO_2_/quartz	2.1294	54	12	6	4.1	2.2
TiO_2_/Si	2.1607	50	10	7	3.9	2.4

The pore radius values determined from the desorption branch of the isotherms are 2.2 and 2.4 nm for the coatings on quartz and silicon substrates, respectively (Table 3). The values of the open porosity of the TiO_2_ coating on the two different substrates determined by EP are very close to each other: 6 and 7% for coatings on quartz and silicon, respectively. These values are approximately 6% (on quartz) and 3% (on Si) less than the total porosity calculated by the Lorentz–Lorenz formula in view of effective refractive indices determined by EP at the zero relative pressure of the adsorptive material (Table 3). The reason for this difference is that one part of the pores is closed or not accessible by the adsorptive molecules.

Considering that ellipsometry is a well-established and reliable technique for determining the thickness and refractive index of thin films, the observed discrepancies in the effective refractive index, and consequently the porosity, of the TiO_2_ coating on a transparent quartz substrate, as derived from various methodologies (see Tables 2 and 3), highlight the potential for greater uncertainty in thin layer optical measurements on transparent substrates. Nonetheless, these findings also indicate that while substrate-type effects^62^ may be present, their magnitude is likely less significant than suggested by the initial thin-layer optical measurement results for the two distinct substrates.

**Figure 4:**
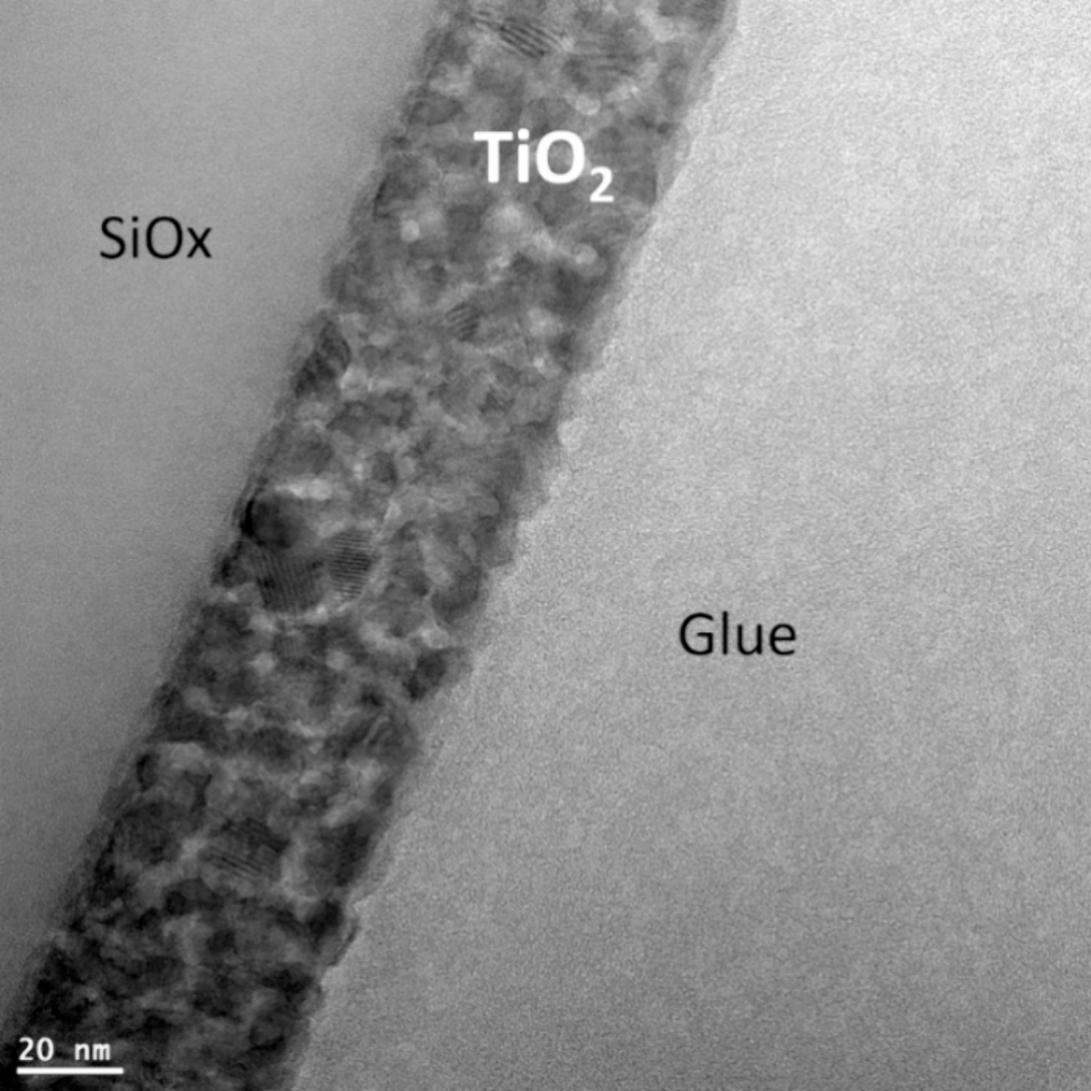
Cross-sectional TEM image of the TiO_2_ sol–gel coating on a silicon substrate.

Figure 4 shows the cross-sectional TEM image of the TiO_2_ coating deposited onto a silicon substrate. It can be seen that the coating is not compact, a granularity is observable that results in a porous structure, in accordance with the results presented in our previous paper.^22^ This porous structure confirms the porosity values determined by different optical methods (UV–vis, SAR, and EP). The thickness of 51 nm determined based on the TEM image is practically identical with the thickness values determined by SAR (52 ± 1 nm) and EP (52 nm) methods for the TiO_2_ coating on a silicon substrate.

### Molecular Dynamics Simulations of Coatings’ Structure Formation

#### Protocol for Computational Synthesis

Here, we describe the computation protocol that we applied to computationally synthesize the porous TiO_2_ layers.

The TiO_2_ gel has a highly complex structure, involving the possible presence of microphases or chains. As here we concentrate on the formation of the TiO_2_ layer, we initiated the computational synthesis process with a TiO_2_ sphere, whose uniform diameter of 4 nm was selected based on the particle diameter (including the solvation shell) of approximately 2.7 nm with a full width at half-maximum of also 2.7 nm determined by DLS measurement. This sphere was carved from a suitably large anatase supercell. The anatase crystal phase was chosen based on the XRD analysis of the TiO_2_ powder samples (see Figure SI4 in the Supporting Information). The simulation is initiated with compact TiO_2_ nanoparticles (solid microphases), which form from the chain-like oligomers and macromolecules initially present in the precursor sol during layer deposition. As a result of evaporation (drying), the system becomes saturated to water, the oligomers lose their solubility which results in the formation of solid microphases. The initially amorphous particles transform into anatase due to the heat treatment.

**Figure 5:**
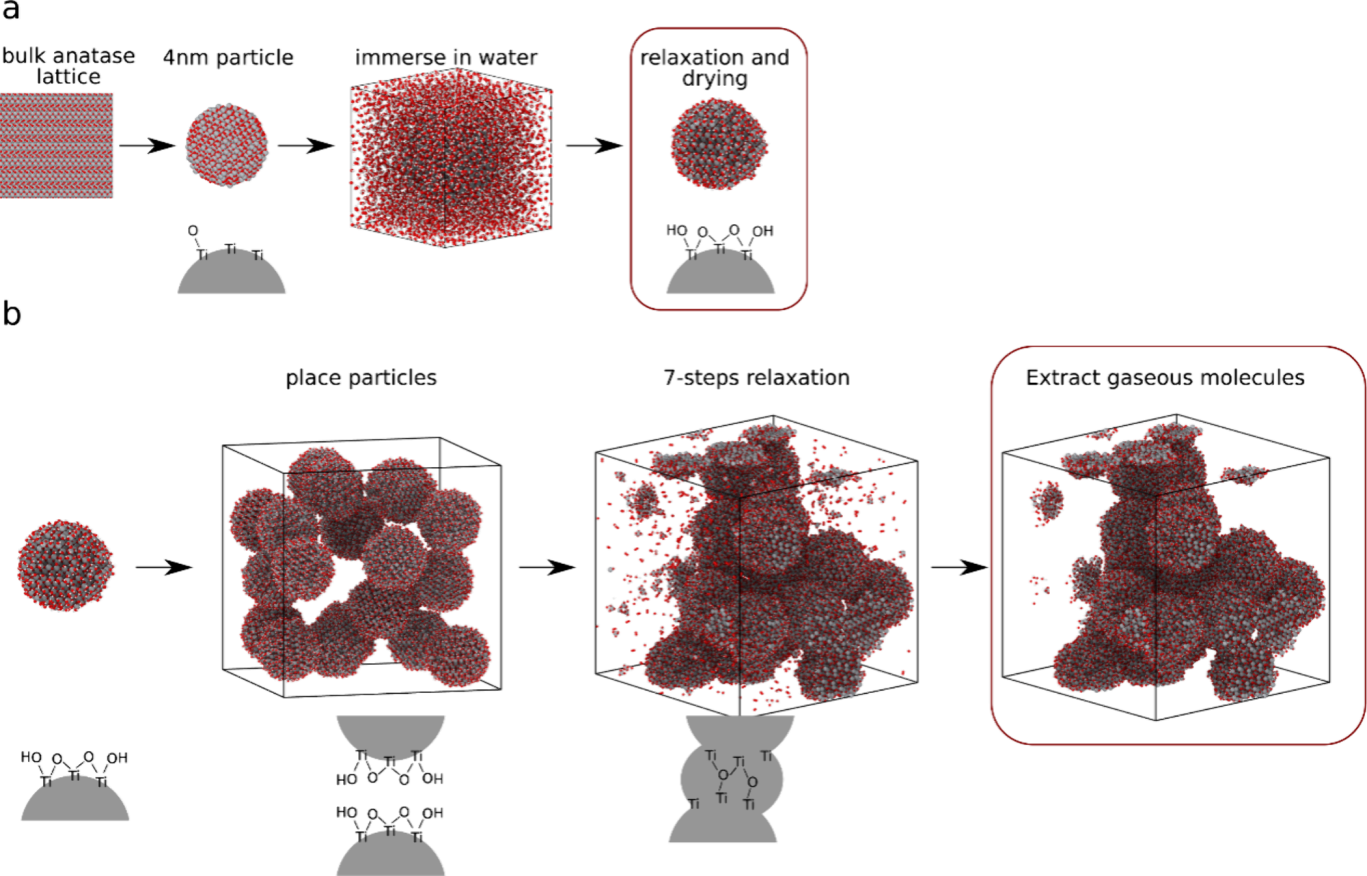
Computational synthesis protocol for porous TiO_2_ layers: (a) Nanoparticle model construction. (b) Porous layer formation.

The carved particle composition was found to be Ti_979_O_1962_, where the ratio of oxygen is 2.004, close to that of bulk TiO_2_. However, since the particle carving was solely based on geometric criteria, the resulting structure contained several low-valent atoms with dangling bonds on the particle surface. In the case of a TiO_2_ particle synthesized in a water-containing environment (i.e., the ethanolic precursor sol also contains a small amount of water for initiating the hydrolysis), the dangling bonds of oxygen and titanium atoms are effectively saturated by hydrogen and hydroxyl groups, respectively. Thus, we immersed the particle in water to allow for an optimal termination and relaxation of the surface. A similar strategy has been applied recently to construct realistically hydrated TiO_2_ surfaces.^36^ After optimizing the initial geometry, we carried out a molecular dynamics simulation for 250 ps at constant temperature and pressure of 300 K and 1 atm to relax the structure. The water and the dissociated products are removed subsequently to model solvent evaporation during the drying process. The composition of the resulting nanoparticle was found to be Ti_973_O_2107_H_492_ (indicating a chemical formula of TiO_2.1_H_0.5_), indicating that a limited number (potentially high-energy) of titanium atoms underwent dissociation from the surface, forming compounds of small molecules. The TiO_2.1_ composition revealed by XPS analysis (Table 1) of the TiO_2_ sample before cluster sputtering is in good agreement with and further supports the simulation results. All of the hydrogen atoms present on the surface are hydroxyl groups; thus, the effective composition of the particle can be described as TiO_1.7_(OH)_0.5_. The excess oxygens and hydroxyls are responsible for the appropriate surface termination. This particle has been applied in all subsequent simulations (see Figure 5a). This is because of the termination of the surface titanium atoms with oxygen or OH groups.

Subsequently, 20 such TiO_2_ particles were placed in a simulation box of 13.7 × 13.7 × 13.7 nm, allowing a nontouching distribution. The box was set under periodic boundary conditions in all three dimensions, which is anticipated to be an appropriate model, considering the experimental layer thickness of approximately 50 nm.

During the experimental drying and annealing process, complex chemical and physical processes occur, ultimately resulting in the formation of a porous layer. The time scale of these processes exceeds the capabilities of molecular dynamics simulations. As a result, we have developed a comprehensive protocol to effectively model the most significant processes, termed the “7-step relaxation process”. This approach draws inspiration from the amorphous polymer setup in molecular dynamics. By employing a suitable setup consisting of multiple short molecular dynamics simulations with appropriate temperature and pressure conditions, we can effectively guide the complex structure toward a well-equilibrated state with a realistic geometric structure.^65^ The layer formation (Figure 5b) was modeled using the protocol shown in Table 4. The simulations start with the modeling of the drying process (step 1). To account for the influence of attractive capillary forces in the drying process, we incorporated an external pressure that was applied exclusively during this phase. This is because capillary forces are active only until the evaporation of the liquid compounds is fully accomplished. In order to align with the typically achievable time scales of molecular dynamics simulations, the drying and annealing temperature [K] used in this study is higher than those employed in the experiments. This adjustment enhances the chemical reaction rates to complete the annealing reactions within the typical molecular dynamics time scale. A similar strategy is employed often in molecular dynamics simulations of polymer setups,^36^ and in reactive force field-based combustion simulations.^66^ The annealing temperature for the simulations was chosen to be lower than the point at which the nanoparticle interior starts to soften during the simulations. The drying process involves a 5 °C/min heat up in the experiments, where complex physicochemical processes take place, including the evaporation of the solvent and the compactification of the structure. We note that such a time scale is beyond the capabilities of the molecular dynamics simulations. Thus, here, we utilize an effective model.

**Table 4:** Simulation Protocol for Nanoparticle Synthesis

step	temperature [K]	pressure [atm]	time [ps]
1	800	fixed pressure, as described (see Figure 6)	50
2	800 → 1000	1	12.5
3	1000	1	50
4	1000 → 1200	1	12.5
5	1200	1	50
6	1200 → 300	1	12.5
7	300	1	50
total			237.5

Consequently, the simulations were initiated at a temperature of 800 K and at the specified pressure. Previous studies employing high-temperature annealing molecular dynamics simulations of TiO_2_ have demonstrated that the anatase phase begins to emerge at this temperature.^67^ Also, neck formation during the TiO_2_ nanoparticle sintering molecular dynamics simulations was observed already at 573 K, thus this temperature makes the agglomeration reactions feasible.^40^ Our simulations were initiated from anatase structure nanoparticles, and we did not observe any change in the crystal structure of the particles. These conditions allowed compactification of the structure, as was shown by the reduction of both the cell size and the porosity.

Simulation steps 2–5 (Table 4) model the annealing during the high-temperature treatment of the porous layer. In a similar manner to the drying step, we utilized a higher temperature of 1000 and 1200 K in the simulations compared to the experimental conditions. This elevated temperature facilitated the appropriate reaction progress. The temperatures were incrementally raised between each target.

In steps 6 and 7 (Table 4), the temperature was reduced to 300 K, followed by a final annealing step to alleviate any potential strain induced by the high temperature.

During the drying and annealing process, the structure released water, oxygen, and small titanium compounds, while Ti–O–Ti bonds were formed between the particles. After the final step, the released gaseous compounds were eliminated, resulting in a clean and porous structure.

We further examined the impact of doubling and quadrupling the simulation time for each step on both the calculated porosity values and the simulated TEM images (Table 5). The simulated TEM images show similar, but not identical, distribution of the atoms and also an increase in the porosity. The increased porosity can likely be attributed to the incomplete annealing of the structure during simulation step 1. The longer subsequent heating steps in the simulations enable a greater degree of relaxation, leading to higher porosity values. Nevertheless, using a given simulation protocol (i.e., total simulation time in the present case), the effect of the total pressure should follow the same trend.

**Table 5:**
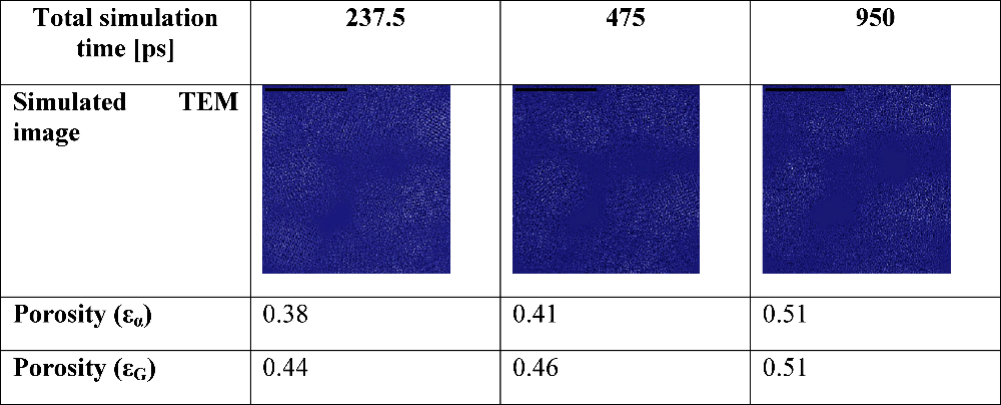
Effect of the Total Simulation Time on the Simulated TEM Images and Porosity (ε_α_: Alpha-Shape Porosity, ε_G_: Gauss-Density-Based Porosity) Values at an External Pressure of 1000 atm^a^

a The length of the scale bar indicates 5 nm.

#### Computational Synthesis of Porous TiO_2_ Layers

The attractive capillary forces are responsible for the shrinkage of the coating during evaporation and for the formation of the pore structure during the drying phase.

While the internal pressure due to the capillary forces can be estimated to range from 180 to 570 atm, we regarded the pressure as a parameter to align the molecular dynamics time scale with that of the experiment and investigated its effect on the pore structure of the coating. The internal pressure due to the capillary forces was approximated by using an average capillary pressure within the layer. The calculations were carried out based on the simplified Laplace–Young equation for spherical geometries, assuming the presence of either pure ethanol (180 atm) or pure water (570 atm), along with complete wetting (for ethanol), or a receding contact angle of 16° (for water)^68^ on the surface of the TiO_2_. The pore radius of 2.4 nm, determined by EP, was used in the calculations. (For further details, please refer to Section 2.7 in the Supporting Information.)

**Figure 6:**
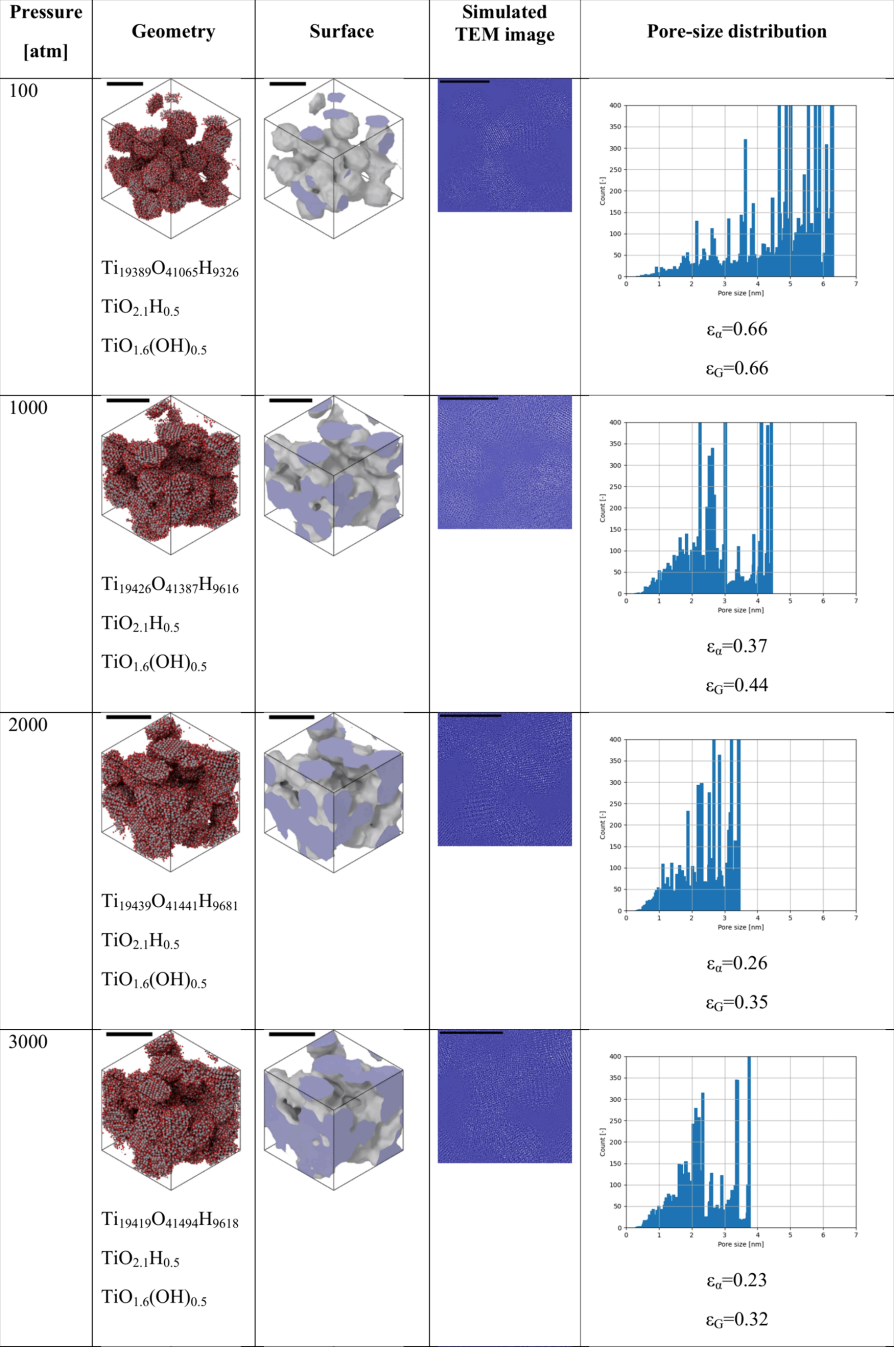

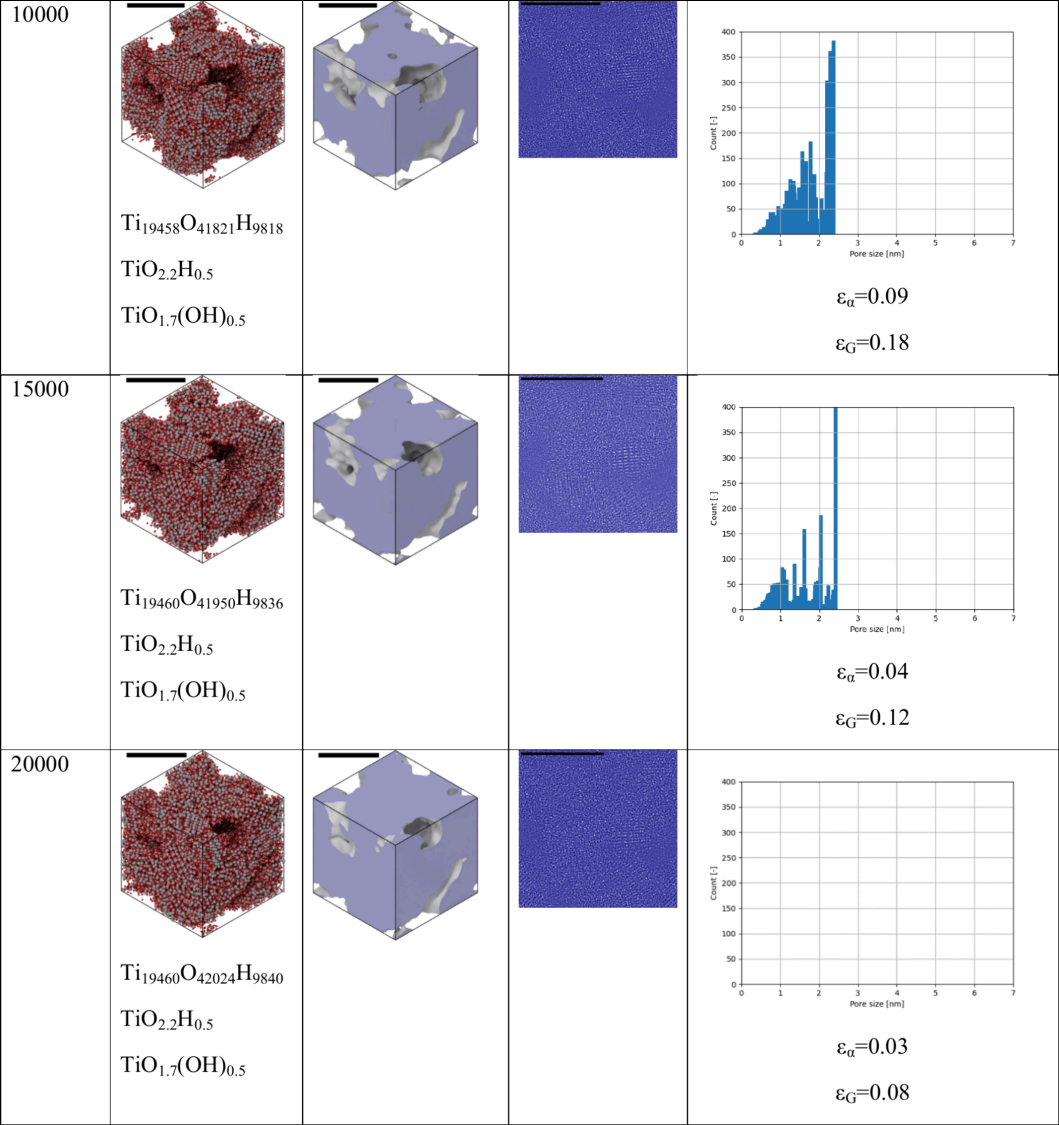
Simulation results using different pressures. The scale bar indicates a length of 5 nm. Geometry was rendered based on the atomic coordinates. Compositions and chemical formulas are indicated below each structure. Surfaces were generated using the alpha-shape method with a probe radius of 3 Å, as implemented in the Ovito code.^51^ All computations are periodic in 3 dimensions; thus, only the simulation cell is shown. Both the alpha-shape (ε_α_) and the Gauss-density-based porosities (ε_G_) are shown.

The simulation results at different pressures ranging from 100 to 20,000 atm are depicted in Figure 6.

It is very visible that the anatase crystal structure of the particles was kept unchanged in the simulations, in line with the selected simulation temperature. Thus, only the particle surfaces are altered by the chemical reactions and by the deformation due to the external pressure. The figure (Figure 6) clearly shows the important effect of the pressure. At a lower pressure of 100 atm, the particles keep their quasi-spherical shape, and point-like joints form between the TiO_2_ particles. The porosity is high, indicating essentially a dried sol state, as indicated also by the very large diameter pores in the pore-size distribution. Nevertheless, the formation of necks between the particles is already evident both from the geometry and also from the simulated TEM image, as the initially separate particles are chemically bonded together through Ti–O bonds in the final state. On the other hand, a higher pressure of 1000 atm leads to a substantial distortion of the particle shapes, and the porosity decreases significantly, in line with the shifting of the pore size distribution toward smaller pore sizes. The neck formation becomes more pronounced with the increased pressure. These trends continue with the further increase of the pressure. However, it is important to note that even at pressures as high as 15,000 atm, certain large-diameter pores still persist. At the highest pressure of 20,000 atm, the connected pore structure diminishes, leaving behind only a few disconnected, small diameter pores. This is well visible both on the geometries and also on the simulated TEM images.

It is interesting to note that the porosities computed using the alpha-shape and the Gauss-density methods follow the same trends; however, due to the definition, the ε_α_ should model the experiments more closely. One possible explanation for the discrepancy between experimental results and simulations (i.e., porosity corresponding to the experimental results is achieved at higher pressure values in the simulations) is that the solid microphases formed during drying are still plastic and, therefore, the coating composed of them is more shrinkable. The simulations clearly reveal that the porosity of sol–gel coatings formed from microphases is influenced not only by capillary forces but also by the plasticity of the particles building up the coating. Hence, by controlling the preparation of precursor sols and the formation of microphases, the porosity can also be regulated. Similarly, drying conditions play a crucial role; under slower drying conditions, the consolidation and hardening of particles may occur at lower pressures, which could result in higher porosity. The plasticity of freshly prepared TiO_2_ layers is also indicated by the observation that such layers can swell in water vapor.^69^

The final compositions clearly show that oxygens are eliminated from the systems. This is because of the formation of Ti–O bonds between the particles, which according to the simulation proceeds not only by the water desorption (formally as Ti–OH + HO–Ti → Ti–O–Ti + H_2_O) but also by direct O_2_ evolution. Interestingly, the formal elimination of hydrogen shows a declining pattern as the pressure increases. Notably, at a pressure as high as 20,000 atm, no hydrogen elimination was observed within the simulation time. Likewise, the formal elimination of titanium from the system follows a similar trend.

These observations clearly demonstrate the impact of external pressure during the drying phase of the simulations. Lower pressures result in a dried, weakly annealed sol state, whereas an extremely high pressure leads to the formation of a mildly porous, polycrystalline state. Therefore, external pressure can be considered a physically motivated parameter, based on the capillary pressure during sol drying, and computationally employed to align the molecular dynamics time scale with experimental conditions. The morphology of the TiO_2_ layer can be tuned by the external pressure, and an appropriate pressure can be selected to align with the experiments. However, it is important to highlight that different pressure ranges result in distinct morphologies. A low pressure of 100 atm leads to a dried, annealed sol state, while pressures ranging from 1000 to 3000 atm yield a porous structure. The structure subjected to approximately 10,000 atm external pressure in the simulations exhibits a comparable porosity to that observed in the bulk material. This finding highlights the role of capillary immersion forces, which can exert a much higher contractive force during sol–gel layer formation than that resulting from capillary pressure.^70^ This similarity in porosity suggests a corresponding similarity in pore structure, making it a suitable computationally synthesized representation of the experimental structure. However, it is important to acknowledge that there are some differences in the pore shapes observed in the simulated and experimental images. Lower pressures yield more connected pore structures, and the one computed at 3000 atm shows the closest agreement with the experiments.

Finally, it is important to note that pressures above 10,000 atm cause the structure to resemble more of a bulk polycrystalline material.

Table 6 shows the bonding analysis of the computationally synthesized TiO_2_ systems at 3000 and 10,000 atm pressures. Bonds were perceived based on distance criteria using the default cutoffs of the Ovito code.

**Table 6:** Bonding Analysis of the Simulation Results at Various Pressures

pressure [atm]	3000	10,000
number of Ti–O bonds [-]	91,506	88,842
number O–H bonds [-]	11,126	11,421
number of O–H bonds on the pore wall [-]	5412	2717
number of O–O bonds [-]	3049	4081
density of O–H bonds on the pore wall [1/nm^2^]	14.5	18.8

The bonding analysis reveals that, as expected, the vast majority of chemical bonds is between the Ti and the O atoms, while a significant number of O–H and, more surprisingly, the O–O bonds also exist. A significant number of O–O bonds are present between the annealed particles. We expect that the formation of O–O bonds can be traced back to simulation reasons, either because their relaxation would need a longer time scale or because the actual compounds (including water, as shown in ref (39)), bound to the surface or present in the atmosphere can suppress their formation or can play an important role in their relaxation during the annealing process.

In line with the observed composition of the computationally synthesized systems, the number of O–H bonds remains relatively constant regardless of the pressure, while specifically on the pore wall, there is a noticeable decrease in the quantity of O–H bonds as the pressure increases. However, it is important to note that as the pore surface decreases with pressure, the density of the O–H bonds on the pore wall actually increases. Surface O–H bonds play an important role in binding dye molecules; thus, the predicted density holds important information, which is difficult to obtain from experiments.

## Conclusions

Here, we present a joint experimental and computational synthesis of titanium-dioxide layers and, in particular, provide simulations for porous-structure formation for the first time. The ReaxFF-based molecular dynamics simulations allowed us to follow the chemical reactions during the drying and the annealing process. The simulations were started from carefully prepared spherical titanium-dioxide nanoparticles with realistic surface termination. The effect of the capillary forces in the drying phase was taken into account by an appropriately selected external pressure in the simulations. Our findings demonstrate that the selection of an appropriate pressure parameter can effectively tune the model to align with experimental results. This pressure parameter is physically motivated and serves as a valuable tool for adjusting the model to match the experimental observations. The simulations yielded pore structures for TiO_2_ with realistic geometries, composition, and also with partial OH termination. However, we highlight that the current version of the model neglects the effect of the substrate, which can influence the pore structure, and it is also clear that the modeling of the local chemical bond formation between the particles needs further improvement.

Thus, here we showed both the usefulness of the model to computationally synthesize nanoporous TiO_2_, and also its limitations, what must be taken into account during the interpretation of the results, and should be investigated.

## Supporting Information Available

Equations used in fitting procedures for UV–vis transmittance curves and reflectance curves, Lorentz–Lorenz equation for porosity calculation; AFM images and additional details about the AFM results; representative size distribution function of the precursor sol measured by DLS; XRD patterns of the powder samples; photographs of the samples; XPS spectra; reflectance curves of the samples taken by SAR; and additional details regarding the estimation of the internal pressure within the layers induced by capillary forces (PDF)
