# Supplementary material for: Experimental and Computational Synthesis of TiO2 Sol–Gel Coatings
Source: Langmuir. 2025 Jan 2;41(1):704–18. doi: 10.1021/acs.langmuir.4c03959 (PMC11736836; doi:10.1021/acs.langmuir.4c03959)
Supplement: Supplementary file 1 — la4c03959_si_001.pdf [file la4c03959_si_001.pdf]

Supporting Information

Experimental and computational synthesis of TiO_2 sol-gel coatings

Emőke Albert[†], *Péter Basa*[‡], *Bálint Fodor*[‡], *Zsófia Keresztes*[§], *János Madarász*[‖], *Péter Márton*[†], *Dániel Olasz*[⊥], *Adél Sarolta Rácz*[⊥], *György Sáfrán*[⊥], *Tamás Szabó*[§], *Borbála Tegze*[†], *Tibor Höltzl*[*,#,∇], *Zoltán Hórvölgyi*[*,†]

[†]Department of Physical Chemistry and Materials Science, Budapest University of Technology and Economics, Műegyetem rkp. 3, 1111 Budapest, Hungary

[*]E-mail: horvolgyi.zoltan@vbk.bme.hu

[‡]Semilab Semiconductor Physics Laboratory Co. Ltd., Prielle Kornélia utca 2, 1117 Budapest, Hungary

[§]HUN-REN Research Centre for Natural Sciences, Institute of Materials and Environmental Chemistry, Magyar tudósok körútja 2, 1117 Budapest, Hungary

[‖]Department of Inorganic and Analytical Chemistry, Budapest University of Technology and Economics, Műegyetem rkp. 3, 1111 Budapest, Hungary

[⊥]HUN-REN Centre for Energy Research, Institute of Technical Physics and Materials Science, Konkoly-Thege Miklós út 29-33, 1121 Budapest, Hungary

[#]HUN-REN Computation Driven Chemistry Research Group, Department of Inorganic and Analytical Chemistry, Budapest University of Technology and Economics, Műegyetem rkp. 3, 1111 Budapest, Hungary

[∇]Nanomaterials Science Group, Furukawa Electric Institute of Technology, Késmárk utca 28/A, 1158 Budapest, Hungary

[*]E-mail: tibor.holtzl@furukawaelectric.com

Table of Contents

1 Characterization methods 3

1.1 Spectrophotometric measurements: determination of thickness and refractive index values of thin coatings on transparent substrates[1] 3

1.2 Scanning angle reflectometry measurements: determination of thickness and refractive index values of thin coatings on non-transparent and transparent substrates[2,3,4] 5

1.3 Lorentz-Lorenz equation for porosity calculation[5] 7

2 Results 8

2.1 Atomic Force Microscopy (AFM) 8

2.2 Dynamic light scattering (DLS) 10

2.3 X-Ray Diffraction (XRD) 11

2.4 Images of the prepared coatings on quartz and silicon substrates 12

2.5 X-Ray Photoelectron Spectroscopy (XPS) 13

2.6 Scanning angle reflectometry (SAR) 15

2.7 Estimation of the internal pressure within the layers induced by capillary forces 16

References 17

1 Characterization methods

1.1 Spectrophotometric measurements: determination of thickness and refractive index values of thin coatings on transparent substrates[1]

The optical properties of the coatings on quartz substrates were determined by a thin-layer optical model (*Hild* method)[1] fitted to the transmittance spectra. The spectra were recorded using a *Hanon i9* UV-Vis spectrophotometer in the wavelength range of 400-1100 nm with 1 nm resolution and a scanning speed of 10 nm/s. The optical model used for samples with one coating at each side of the transparent substrate (in this case on quartz) assumes a homogeneous layer with no light absorption and scattering in the visible wavelength range, and no refractive index gradient along the layer normal. The variables of the single-layer model were the transmittance (T) and the wavelength of the incident light (x) in 0.1 μm units. The iterated parameters were the refractive index (n_0), the dispersion factor (q), and the thickness in 0.1 μm units (d) of the layer, respectively. The model uses the refractive index (p_1) and dispersion factor (p_2) of the quartz substrate as constant values (1.4570 and 0.7, respectively). The transmittance of the system as a function of the parameters are given by Equation SI1:

$$T=\frac{1-\dfrac{\left(1-n_0\sqrt{1+q\left(\frac{1}{x^2}-\frac{1}{40}\right)}\left(1-\frac{2a}{a^2+b^2}\right)\right)^2+\left(\frac{x}{2\pi d}\left(1-\sqrt{\frac{n_0\sqrt{1+q\left(\frac{1}{x^2}-\frac{1}{40}\right)}}{\frac{n_0\sqrt{1+q\left(\frac{1}{x^2}-\frac{1}{40}\right)}}{g}}}\right)-\frac{2\cdot n_0\sqrt{1+q\left(\frac{1}{x^2}-\frac{1}{40}\right)}\,b}{a^2+b^2}\right)^2}{\left(1+n_0\sqrt{1+q\left(\frac{1}{x^2}-\frac{1}{40}\right)}\left(1-\frac{2a}{a^2+b^2}\right)\right)^2+\left(\frac{x}{2\pi d}\left(1-\sqrt{\frac{n_0\sqrt{1+q\left(\frac{1}{x^2}-\frac{1}{40}\right)}}{\frac{n_0\sqrt{1+q\left(\frac{1}{x^2}-\frac{1}{40}\right)}}{g}}}\right)-\frac{2\cdot n_0\sqrt{1+q\left(\frac{1}{x^2}-\frac{1}{40}\right)}\,b}{a^2+b^2}\right)^2}}{1+\dfrac{\left(1-n_0\sqrt{1+q\left(\frac{1}{x^2}-\frac{1}{40}\right)}\left(1-\frac{2a}{a^2+b^2}\right)\right)^2+\left(\frac{x}{2\pi d}\left(1-\sqrt{\frac{n_0\sqrt{1+q\left(\frac{1}{x^2}-\frac{1}{40}\right)}}{\frac{n_0\sqrt{1+q\left(\frac{1}{x^2}-\frac{1}{40}\right)}}{g}}}\right)-\frac{2\cdot n_0\sqrt{1+q\left(\frac{1}{x^2}-\frac{1}{40}\right)}\,b}{a^2+b^2}\right)^2}{\left(1+n_0\sqrt{1+q\left(\frac{1}{x^2}-\frac{1}{40}\right)}\left(1-\frac{2a}{a^2+b^2}\right)\right)^2+\left(\frac{x}{2\pi d}\left(1-\sqrt{\frac{n_0\sqrt{1+q\left(\frac{1}{x^2}-\frac{1}{40}\right)}}{\frac{n_0\sqrt{1+q\left(\frac{1}{x^2}-\frac{1}{40}\right)}}{g}}}\right)-\frac{2\cdot n_0\sqrt{1+q\left(\frac{1}{x^2}-\frac{1}{40}\right)}\,b}{a^2+b^2}\right)^2}} \tag{SI1}$$

where a and b are defined by Equation SI2 and SI3.

$$a = 1 + \frac{\left(n_0\sqrt{1+q\left(\frac{1}{x^2}-\frac{1}{40}\right)}\right)^2 - \left(p_1\sqrt{1+p_2\left(\frac{1}{x^2}-\frac{1}{40}\right)}\right)^2}{\left(n_0\sqrt{1+q\left(\frac{1}{x^2}-\frac{1}{40}\right)} - p_1\sqrt{1+p_2\left(\frac{1}{x^2}-\frac{1}{40}\right)}\right)^2} \cdot \cos\left(\frac{4\pi d n_0\sqrt{1+q\left(\frac{1}{x^2}-\frac{1}{40}\right)}}{x}\right) \tag{SI2}$$

$$b = \frac{\left(n_0\sqrt{1+q\left(\frac{1}{x^2}-\frac{1}{40}\right)}\right)^2 - \left(p_1\sqrt{1+p_2\left(\frac{1}{x^2}-\frac{1}{40}\right)}\right)^2}{\left(n_0\sqrt{1+q\left(\frac{1}{x^2}-\frac{1}{40}\right)} - p_1\sqrt{1+p_2\left(\frac{1}{x^2}-\frac{1}{40}\right)}\right)^2} \cdot \sin\left(\frac{4\pi d n_0\sqrt{1+q\left(\frac{1}{x^2}-\frac{1}{40}\right)}}{x}\right) \tag{SI3}$$

1.2 Scanning angle reflectometry measurements: determination of thickness and refractive index values of thin coatings on non-transparent and transparent substrates[2,3,4]

The optical model used for the samples with one coating on non-transparent (in this case silicon) substrate also assumes a homogeneous layer with no absorption and no refractive index gradient along the layer normal. The variables of the model were the reflected intensity (I) and the angle of incidence (x) in radians. The iterated parameters were the refractive index (n) and the thickness (d) of the coating and a correction factor (h) for better fitting (this can vary in the 1 ± 0.0001 range). The model uses the refractive index (n_s) of the substrate (in case of silicon substrate $n_s = 3.883$).

The intensity of the reflected light as a function of the parameters is given by Equation SI4:

$$I = h \cdot \frac{\left(\frac{\cos x - \frac{\sqrt{n^2 - \sin^2 x}}{n^2}}{\cos x + \frac{\sqrt{n^2 - \sin^2 x}}{n^2}}\right)^2 + \left(\frac{\frac{\sqrt{n^2 - \sin^2 x}}{n^2} - \frac{\sqrt{n_s^2 - \sin^2 x}}{n_s^2}}{\frac{\sqrt{n^2 - \sin^2 x}}{n^2} + \frac{\sqrt{n_s^2 - \sin^2 x}}{n_s^2}}\right)^2 + 2\frac{\frac{\sqrt{n^2 - \sin^2 x}}{n^2} - \frac{\sqrt{n_s^2 - \sin^2 x}}{n_s^2}}{\frac{\sqrt{n^2 - \sin^2 x}}{n^2} + \frac{\sqrt{n_s^2 - \sin^2 x}}{n_s^2}} \cdot \frac{\cos x - \frac{\sqrt{n^2 - \sin^2 x}}{n^2}}{\cos x + \frac{\sqrt{n^2 - \sin^2 x}}{n^2}} \cdot \cos\left(2d\sqrt{n^2 - \sin^2 x}\right)}{1 + \left(\frac{\cos x - \frac{\sqrt{n^2 - \sin^2 x}}{n^2}}{\cos x + \frac{\sqrt{n^2 - \sin^2 x}}{n^2}}\right)^2 \cdot \left(\frac{\frac{\sqrt{n^2 - \sin^2 x}}{n^2} - \frac{\sqrt{n_s^2 - \sin^2 x}}{n_s^2}}{\frac{\sqrt{n^2 - \sin^2 x}}{n^2} + \frac{\sqrt{n_s^2 - \sin^2 x}}{n_s^2}}\right)^2 + 2\frac{\frac{\sqrt{n^2 - \sin^2 x}}{n^2} - \frac{\sqrt{n_s^2 - \sin^2 x}}{n_s^2}}{\frac{\sqrt{n^2 - \sin^2 x}}{n^2} + \frac{\sqrt{n_s^2 - \sin^2 x}}{n_s^2}} \cdot \frac{\cos x - \frac{\sqrt{n^2 - \sin^2 x}}{n^2}}{\cos x + \frac{\sqrt{n^2 - \sin^2 x}}{n^2}} \cdot \cos\left(2d\sqrt{n^2 - \sin^2 x}\right)} \tag{SI4}$$

In the case of studying transparent substrates (in this case quartz) with a coating on both sides with scanning angle reflectometry, it was necessary to take into account the backside reflection of the laser light. For this, a modified optical model was used, which also assumes homogeneous layers with no absorption and no refractive index gradient. This model was the following:

$$I = h \cdot \frac{2r}{1+r} \tag{SI5}$$

where I is the reflected intensity, r is the second factor of the product on the right side of Equation S14 (the large fraction), and h is the correction factor described above.

1.3 Lorentz-Lorenz equation for porosity calculation[5]

For calculating the porosity of the mesoporous TiO_2 coatings, the Lorenz-Lorentz formula[5] (Equation SI6) was used:

$$\frac{n_{eff}^2 - 1}{n_{eff}^2 + 2} = \alpha \frac{n_1^2 - 1}{n_1^2 + 2} + (1-\alpha) \frac{n_2^2 - 1}{n_2^2 + 2} \tag{SI6}$$

where n_{eff} is the effective refractive index of the porous material (calculated from the fitting discussed above) n_1 and n_2 are the refractive indices of the two components and α is the volume fraction of component 1. It was assumed in the calculations that the refractive index of TiO_2 is 2.400 and the refractive index of air is 1. If the TiO_2 is considered as component 1, α gives the porosity directly.

2 Results

2.1 Atomic Force Microscopy (AFM)

The **C_0/10** sample was found to be smooth, the RMS roughness measured over a 10 × 10 μm^2 area was S_q = 455.5 pm, the mean roughness S_a = 353.2 pm. A randomly selected cross-section profile (1024 points) is shown below without any averaging.

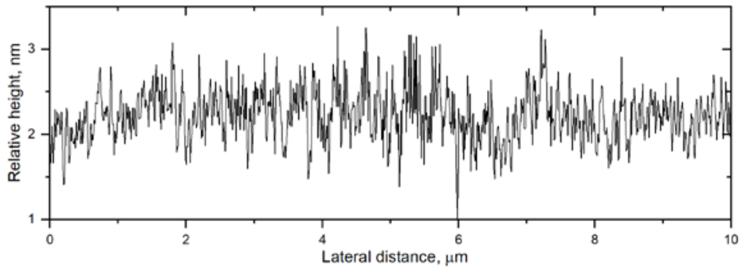

Figure SI1: AFM cross-section profile (1024 points) of the **C_0/10** sample

The corresponding phase image of the **C_0/10** sample (image (a) in the Figure below) is homogeneous. In a window of **400 × 400 nm^2**, fine granular character can be observed. On the **C_0/100** sample, “holes” and “craters” appear on the surface. A typical landscape can be seen in the image (image (b) in the Figure below, **1 × 1 μm^2**). At the bottom of the holes, granular surface structure can be observed (not shown). The phase images do not refer to any material inhomogeneity. In the case of the **C_0/200** sample, the lateral size of the holes and craters became larger. The remaining walls between the expanded holes form mesh-like structure (image (c) in the Figure below, **1 × 1 μm^2**).

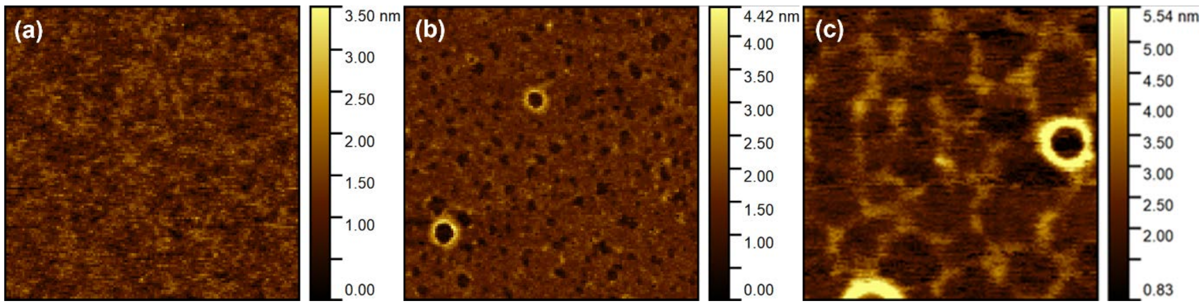

Figure SI2: AFM images of the samples deposited on Si-substrates by spin-coating from the precursor sols diluted with ethanol to **C_0/10** (a), **C_0/100** (b), and **C_0/200** (c) concentrations. (a): 10× 10 μm^2, (b): 1 × 1 μm^2, (c): 1 × 1 μm^2 (C_0 is the initial concentration of the precursor sol).

Based on the AFM images, it can be concluded that the dilution of the precursor sol influences the structure due to evaporation; however, individual particles are not visible.

2.2 Dynamic light scattering (DLS)

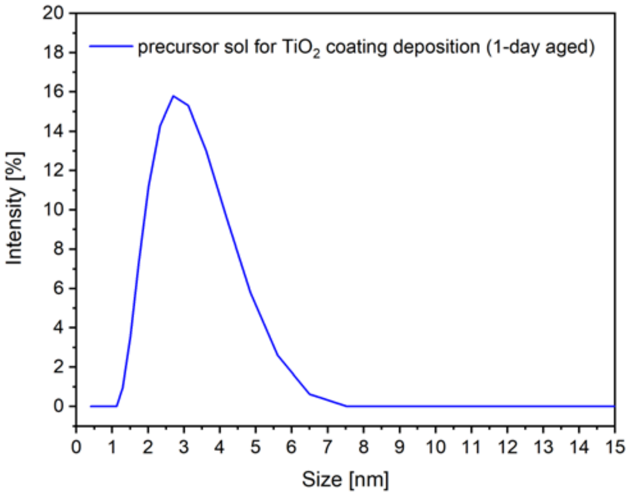

Figure SI3: Representative size distribution function of the 1-day aged TiO_2 precursor sol obtained by DLS studies.

2.3 X-Ray Diffraction (XRD)

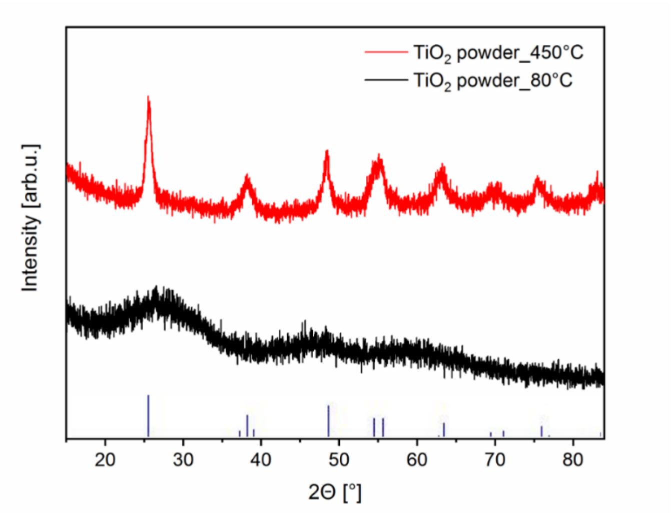

Figure SI4: XRD patterns of the TiO_2 powder samples prepared from the 1-day aged precursor sol. One of the samples was dried at 80 °C (black pattern), the other sample was heat-treated at 450 °C (red pattern). The positions of the standard reflections for anatase [PDF 98-000-9852] are also shown as vertical bars at the bottom. The diagram is shifted along the Intensity axis for better visibility.

2.4 Images of the prepared coatings on quartz and silicon substrates

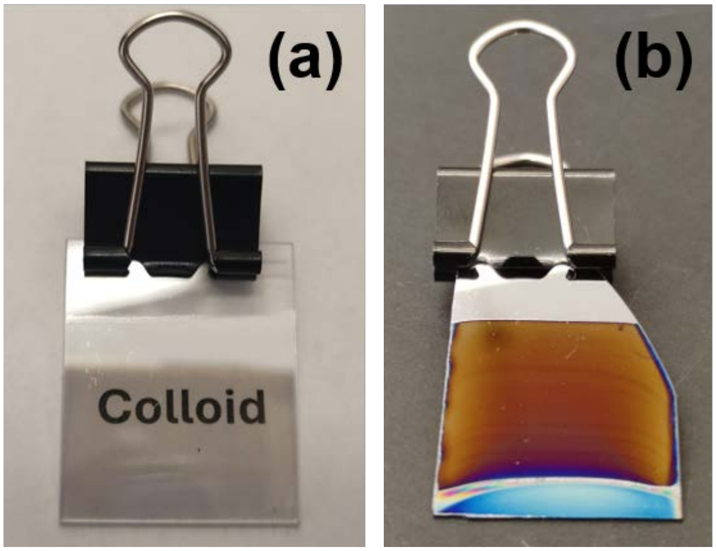

Figure SI5: Images of the TiO_2 coatings on quartz (a), and silicon (b) substrates. The transparency of the sample on quartz substrate is well observable in image (a): the text on the paper placed under the sample can be seen clearly. The prepared TiO_2 coatings exhibited homogeneity on both quartz and silicon substrates (The extended, thicker region of the coating at the bottom of the silicon substrate is attributed to the edge effect of the substrate.).

2.5 X-Ray Photoelectron Spectroscopy (XPS)

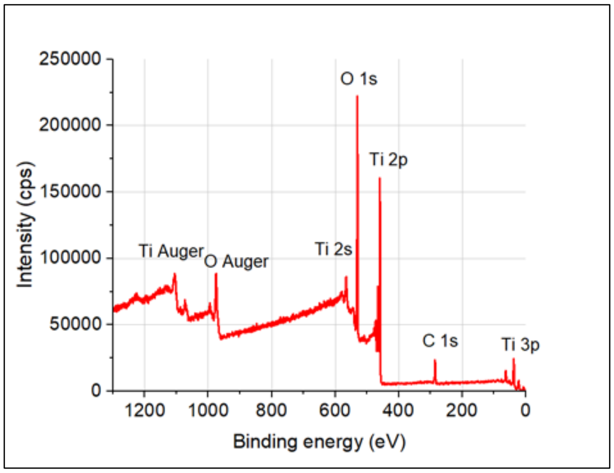

Figure SI6: XPS survey spectrum of the of the TiO_2 coating on silicon substrate (before cluster sputtering).

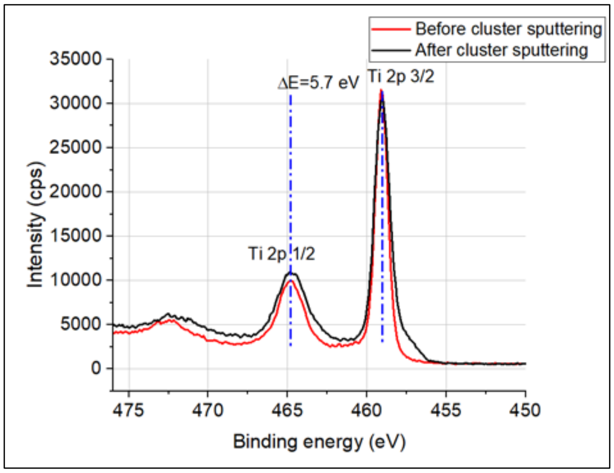

Figure SI7: XPS high resolution Ti2p spectrum of the TiO_2 coating on silicon substrate before, and after cluster sputtering.

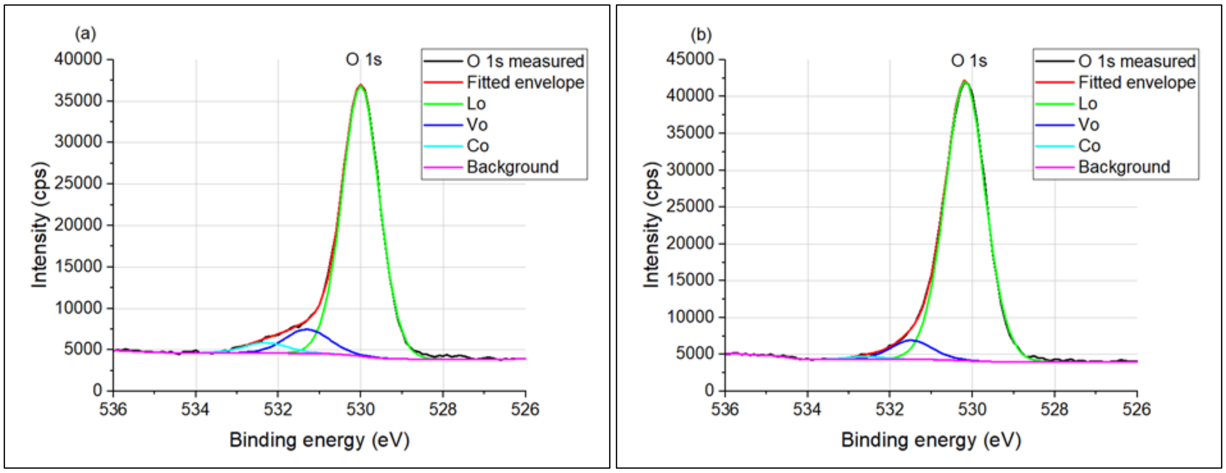

Figure SI8: XPS high resolution O 1s spectrum of the TiO_2 coating on silicon substrate before argon cluster sputtering (a), and after argon cluster sputtering (b). L_O stands for lattice oxygen, namely the Ti-O bonds in TiO_2,while V_O to oxygen vacancy/defects, and C_O belongs to loosely adsorbed, dissociated oxygen or OH species.

2.6 Scanning angle reflectometry (SAR)

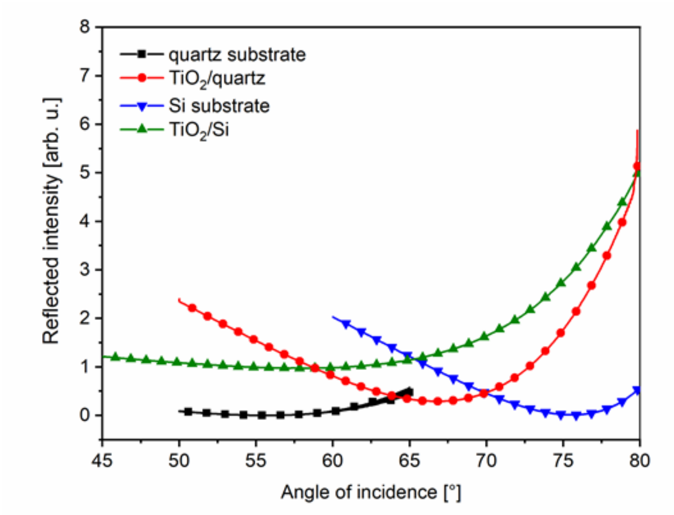

Figure SI9: Smoothed reflectance curves of TiO_2 coatings on quartz and silicon substrates, together with those of their bare substrates

2.7 Estimation of the internal pressure within the layers induced by capillary forces

The internal pressure in the lyogels immediately after the layer deposition was approximated by an average capillary pressure (P_c) within the layer.

The calculation was based on the simplified Laplace-Young equation (Equation SI7) for spherical geometries, assuming the presence of pure ethanol only (i.e. the dispersion medium, surface tension of ethanol at 25 °C: γ = 21.82 mN/m), and complete wetting (contact angle: θ = 0°). The pore radius (r) of 2.4 nm, determined by ellipsometric porosimetry (see Table 3 in the Manuscript) was used in the calculations.

The capillary pressure is given by the Equation SI7:

$$P_c = \frac{2\gamma}{r} \cdot cos\theta \tag{SI7}$$

Considering that for θ = 0° cos(θ) = 1, the expression simplifies to:

$$P_c = \frac{2\gamma}{r} = \frac{2 \cdot 21.82\ \mathrm{mN/m}}{2.4\ nm} = 18.1833 \cdot 10^6\ \frac{N}{m^2} = 179.5\ atm \tag{SI8}$$

For pure water (with a surface tension of γ = 71.99 mN/m at 25°C), and considering a receding contact angle of θ = 16°, where cos(θ) = 0.96, the expression is:

$$P_c = \frac{2\gamma}{r} \cdot cos\theta = \frac{2 \cdot 71.99\ \mathrm{mN/m}}{2.4\ nm} \cdot 0.96 = 57.5920 \cdot 10^6\ \frac{N}{m^2} = 568\ atm \tag{SI9}$$

References

(1) Hild, E.; Deák, A.; Naszályi, L.; Sepsi, Ö.; Ábrahám, N.; Hórvölgyi, Z. Use of the Optical Admittance Function and Its WKB Approximation to Simulate and Evaluate Transmittance Spectra of Graded-Index Colloidal Films. *J. Opt. Pure Appl. Opt.* **2007**, *9* (10), 920–930. https://doi.org/10.1088/1464-4258/9/10/023.

(2) Hild, E.; Seszták, T.; Völgyes, D.; Hórvölgyi, Z. Characterisation of Silica Nanoparticulate Layers with Scanning-Angle Reflectometry. In *From Colloids to Nanotechnology*; Zrínyi, M., Hórvölgyi, Z. D., Eds.; Springer Berlin Heidelberg: Berlin, Heidelberg, 2004; pp 61–67. https://doi.org/10.1007/978-3-540-45119-8_11.

(3) Deák, A.; Hild, E.; Kovács, A. L.; Hórvölgyi, Z. Characterisation of Solid Supported Nanostructured Thin Films by Scanning Angle Reflectometry and UV-Vis Spectrometry. *Mater. Sci. Forum* **2007**, *537–538*, 329–336. https://doi.org/10.4028/www.scientific.net/MSF.537-538.329.

(4) Mann, E. K.; Van Der Zeeuw, E. A.; Koper, G. J. M.; Schaaf, P.; Bedeaux, D. Optical Properties of Surfaces Covered with Latex Particles: Comparison with Theory. *J. Phys. Chem.* **1995**, *99* (2), 790–797. https://doi.org/10.1021/j100002a049.

(5) Heller, W. Remarks on Refractive Index Mixture Rules. *J. Phys. Chem.* **1965**, *69* (4), 1123–1129. https://doi.org/10.1021/j100888a006.
